# Tumour regression predicts better response to interferon therapy in melanoma patients: a retrospective single centre study

**DOI:** 10.1097/CMR.0000000000000935

**Published:** 2023-11-13

**Authors:** Noémi E. Mezőlaki, Eszter Baltás, Henriette L. Ócsai, Anita Varga, Irma Korom, Erika Varga, István B. Németh, Erika G. Kis, János Varga, Ádám Kocsis, Rolland Gyulai, Mátyás Bukva, Lajos Kemény, Judit Oláh

**Affiliations:** 1 Department of Dermatology and Allergology, Albert Szent-Györgyi Health Center, University of Szeged, Hungary

**Keywords:** interferon, immunotherapy, melanoma, ulceration, regression

## Abstract

We hypothesise that regression may have an impact on the effectiveness of adjuvant IFN therapy, based on its role in the host immune response. Our purpose is to investigate regression and ulceration as prognostic factors in case of interferon-alpha (IFN)-treated melanoma patients. We followed 357 IFN-treated melanoma patients retrospectively, investigating progression-free survival (PFS) and overall survival (OS) depending on the presence of ulceration and regression. A Kaplan–Meier analysis was performed, and we used a Cox regression analysis to relate risk factors. The survival function of the Cox regression was used to measure the effect of regression and ulceration on PFS and OS depending on the Breslow thickness (T1–T4) of the primary tumour. Regression was significantly positively related to PFS (*P* = 0.0018, HR = 0.352) and OS (*P* = 0.0112, HR = 0.380), while ulceration showed a negative effect (PFS: *P* = 0.0001, HR = 2.629; OS: *P* = 0.0003, HR = 2.388). They influence survival independently. The most favourable outcome was measured in the regressed/non-ulcerated group, whereas the worse was in the non-regressed/ulcerated one. Of risk factors, Breslow thickness is the most significant predictor. The efficacy of regression is regardless of Breslow thickness, though the more favourable the impact of regression was in the thicker primary lesions. Our results indicate that regression is associated with a more favourable outcome for IFN-treated melanoma patients, whereas ulceration shows an inverse relation. Further studies are needed to analyse the survival benefit of regression in relation to innovative immune checkpoint inhibitors.

## Introduction

Primary cutaneous melanoma is responsible for most skin cancer-related deaths; however, it accounts for only approx 3% of all malignant skin tumours [1]. This aggressive cancer is of enormous significance, as its incidence is still rapidly growing worldwide.

The treatment of melanoma has seen a paradigm shift both in metastatic and adjuvant therapy. Innovative targeted therapy and immune checkpoint inhibitor treatments have significantly improved the life prospects of melanoma patients over the past ten years. Nowadays, the above new drugs can be used not only in metastatic melanoma but also as adjuvant treatment in the case of advanced primary tumours. However, more and more data indicate that immunotherapies used as primary treatment are more beneficial for overall survival than targeted therapy, even in patients with BRAF-mutant metastatic melanoma. The data regarding adjuvant treatments are relatively immature.

According to the current funding protocol in Hungary, BRAF mutant patients can only receive targeted treatment both as a first-line and as an adjuvant therapy.

The local regulation led our thinking in the direction of investigating whether there is a simple histological marker apart from the mutation that could potentially influence the choice of primary therapy for our patients, both from a professional and financial point of view. In our clinical practice, we have observed several cases in our patients with metastatic melanoma, where regression of the primary melanoma, even in the presence of a huge tumour mass, led to permanent complete remission with PD-1 inhibitory immunotherapy. These above observations raised the idea that it is worthwhile to examine the late results of our patients with regression melanoma who received interferon alfa adjuvant treatment due to the overall survival from the era when only interferon therapy was used as standard treatment.

In this study, we focus on two histological features of primary melanoma, ulceration and regression, to investigate their impact on overall survival (OS) and progression-free survival (PFS) in IFN-treated patients. Overall survival refers to the time which begins at diagnosis and up to the time of death. Progression-free survival refers to the amount of time between when a treatment for cancer begins, and when cancer progresses or death occurs.

Ulceration is a well-defined prognostic factor for patients with cutaneous melanoma, which is associated with poor prognosis and an increased risk of mortality [2–4]. This implication is presumably related to increased tumour thickness, mitotic rate and vascular invasion in the primary tumour [5–7]. The impact of ulceration on interferon-treated patients has already been investigated. Kelly *et al*. performed a post hoc analysis (the Sunbelt Melanoma Trial), in which they support the conclusion that ulceration predicts improved response to adjuvant IFN therapy [8].

In contrast to ulceration, the prognostic implication of regression in primary melanoma has been debated in recent decades and interpreted in contradictory ways, in part because inconsistent histological criteria are used in prognostication studies. Karina A. *et al*. summarise in their review these controversies, and find that no universally accepted scheme exists today [9]. According to the College of American Pathologists, characteristic histological features of regression are replacement of tumour cells by lymphocytic inflammation, attenuation of the epidermis, and non-laminated dermal fibrosis with inflammatory cells, melanophagocytosis, and telangiectasia [10]. Our pathologists' colleagues were attentive to the above criteria while examining the histological sections.

The aetiology of spontaneous regression is multifactorial. It is generally considered to be the outcome of host immune-mediated responses, including the activation of CD8-positive cytotoxic T lymphocytes (CTLs), CD4-positive T lymphocytes and Th1 cytokines [11]. Previously, the presence of regression was considered to be a negative prognostic factor, as the disappearance of tumour cells may lead to an underestimation of initial melanoma thickness, thus negatively affecting proper staging [12–15]. Some authors have suggested that patients with histological regression have an increased risk of developing metastasis [15,16]. In contrast, others have reported that this feature was significantly associated with a better prognosis [17–20]. There is no data in the literature on the prognostic factor of spontaneous regression of primary melanoma in IFN treatment. IFN plays a key role in activation of cellular immunity and subsequently in stimulation of an antitumour immune response [21].

It has been thought-provoking for us to have treated a number of melanoma patients in our clinical practice, where we have detected significant regression in the primary tumour both clinically and histopathologically and experienced an unusually favourable clinical response beyond the treatments administered and despite the large tumour volume. A standard team involving a surgeon, oncodermatologist and dermopathologist has participated in the treatment of melanoma patients at our clinics for three decades. We, therefore, thought it would be worthwhile to conduct a retrospective re-assessment of the clinical and histopathological data on our melanoma patients treated with adjuvant interferon.

Hence, our hypothesis that there may be a link is based on regression being a phenomenon of a host immune-mediated response, which could enhance the efficacy of the immune-modulating effect of IFN therapy.

## Materials and methods

### Clinicopathologic characteristics and patient grouping

In this retrospective, single-centre study, clinical and histopathological data from 428 melanoma patients were registered. After the study was approved by the Ethics Committee and Institutional Review Board at the University of Szeged, Hungary (permission number: MEL-RETRO-001:3521, 40/2015 SZTE), all the records were collected from the Medsol medical database, regarding to the current GDPR. Selection criteria were immunotherapy with INF, independently of subtype (IFNa2a or IFNa2b) and dosing (sc. 3 times/week 3–10 Million International Units). All the patients started to receive IFN therapy in an adjuvant setting from 1 January 2000 to 31 December 2018 in the Department of Dermatology and Allergology, University of Szeged, Hungary. The median follow-up was 112 months in case of OS and 102 months in case of PFS. Patients were excluded with unknown primary tumour or multiple primary melanomas. Eventually, data analyses were performed from a total of 357 IFN-treated melanoma patients. The patients’ ethnic background is middle-Europian (Hungarian), and they belong to ECOG performance status of 0.

Before the adjuvant IFN therapy, all the patients underwent wide local excision of their primary melanomas and a sentinel lymph node biopsy (SLN) was performed in 97.4% of them using a double labelling technique. In nine cases, as a consequence of technical difficulties associated with SLN or considering the patient’s aspect, the lymphatic mapping was not carried out. The histological features were evaluated by our experienced dermatopathologists based on sections from primary melanomas and serial sections of sentinel nodes stained with haematoxylin/eosin and immunohistochemistry (HMB45 and Melan A). They investigated the presence of lymphocytic inflammation, fibrosis, melanophagocytosis, and telangiectasia. Radical regional lymph node dissection was performed in the group of patients with positive SLN or clinically detectable metastatic lymph nodes. All the patients received interferon-alpha (IFN) as an adjuvant therapy after locoregional surgical interventions were completed, as they were considered to be at high risk of recurrence.

In the statistical analyses, patients were grouped in two ways: according to the presence or absence of ulceration and regression (Grouping 1: NR: non-regression group; R: regression group; NU: non-ulceration group; U; ulceration group) and according to the two histopathological characteristics simultaneously (Grouping 2: NR/NU: non-regression and non-ulceration group; R/NU: regression and non-ulceration group; NR/U: non-regression and ulceration group; R/U: regression and ulceration group). 16 patients were excluded from Grouping 2 because their ulceration status was unknown. See Table 1. for patient cohort data (both groupings).

**Table 1:** Patient cohort

		Number of patients	Male		Female		Age	
			Count	%	Count	%	Mean	Median
Grouping 1	NR	279	117	41.94	162	58.06	54.3	51
	R	78	40	51.28	38	48.72	54.4	55
	NU	177	81	45.76	96	54.24	47.1	47
	U	160	64	40.00	96	60.00	53.2	54
Grouping 2	Excluded	16	8	50.00	8	50.00	54.8	54
	NR/NU	134	57	42.54	77	57.46	51.2	44
	R/NU	43	24	55.81	19	44.19	53.8	57
	NR/U	136	55	40.44	81	59.56	53.7	54
	R/U	28	13	46.43	15	53.57	56.1	55

### Statistical method

A Kaplan–Meier analysis with the logrank approach was used to compare OS and PFS functions and to calculate HR values. HR values are presented with 95% confidence intervals. The effects of different predictor variables on OS and PFS were examined using the Cox proportional hazards model with Wald’s forward stepwise selection method. Independence between categorical variables was tested with the Chi-square test. Column proportions were compared using the pairwise z-test with the Bonferroni correction. The statistical analyses were carried out with IBM SPSS 25 and GraphPad Prism 8 software. *P *< 0.05 was considered significant.

## Results

### Regression and ulceration are independently occurring histopathological features

In order to test the independent occurrence of ulceration and regression, we stratified patients (n = 330) according to the presence or absence of ulceration and regression. 39.7% of the melanoma patients showed neither regression nor ulceration, 13.03% regressed but did not ulcerate, 49% had ulcerated but not regressed melanoma, and 8.2% showed signs of both regression and ulceration at the same time. Based on the Chi-square test, no relation was found between the incidence of ulceration and regression. Regression and ulceration occur independently of each other.

### Regression and ulceration have opposite effects on OS and PFS in melanoma patients treated with IFN

**Fig. 1:**
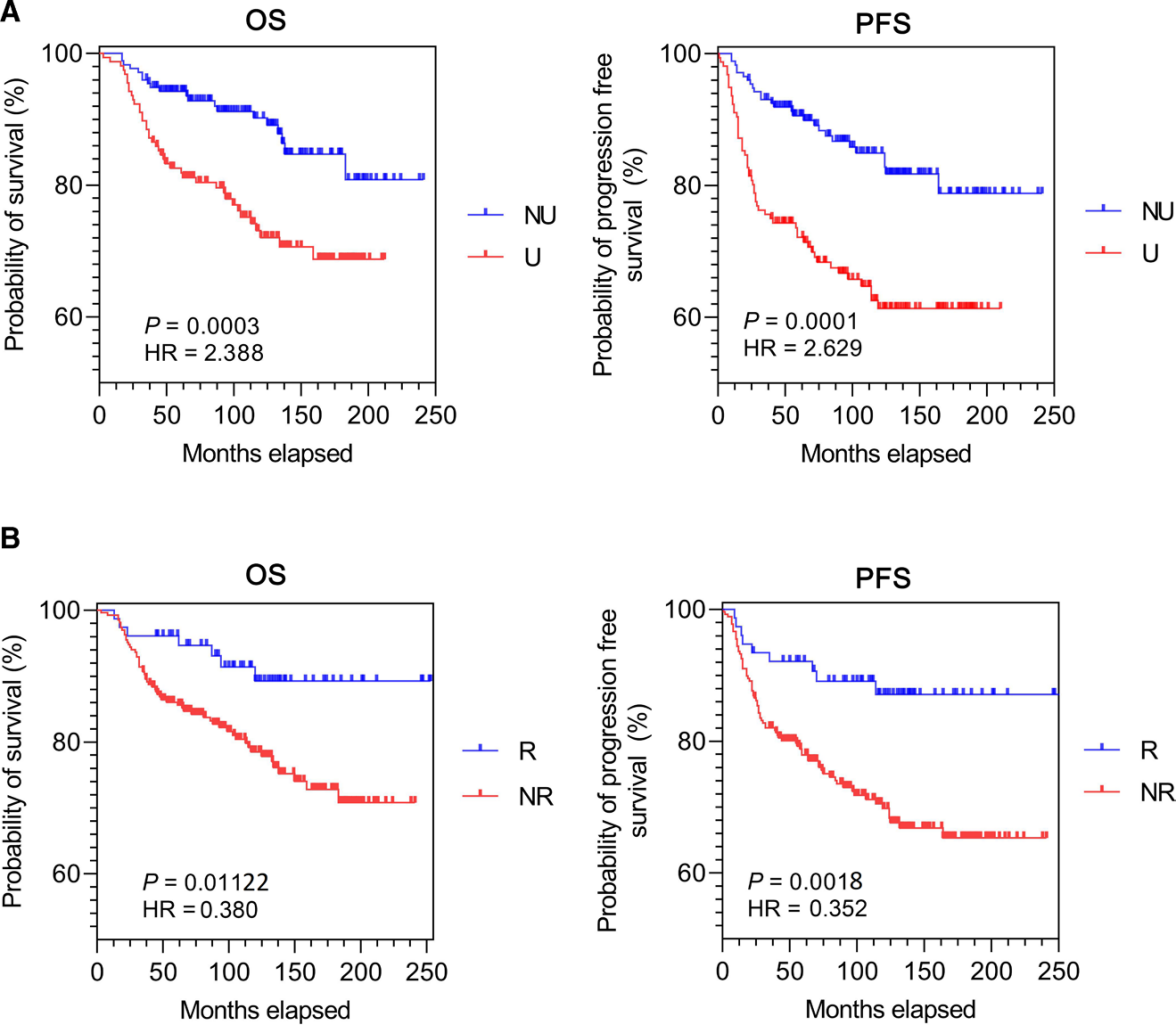
Kaplan–Meier analysis for OS and PFS in the NU, U, R and NR groups. Panels a and b show the OS and PFS, respectively. HR, hazard ratio; NR, non-regression; NU, non-ulceration; OS, overall survival; *P, P*-value; PFS, progression-free survival; R, regression; U, ulceration.

Ulceration was found to be a negative prognostic factor associated with decreased OS (HR = 2.388; *P* = 0.0003) and PFS (HR = 2.629; *P* = 0.0001) among the melanoma patients treated with IFN (Fig. 1). In the NU and U groups, the 5-year OS rate was measured as 94 and 83%, respectively, and the PFS rate was 91% and 72%, respectively. In contrast, the presence of spontaneous regression in primary melanoma is presumed to be a positive prognostic factor due to the increase of both OS (HR = 0.380; *P* = 0.01122) and PFS (HR = 0.352; *P* = 0.0018) in the group of patients treated with IFN. In the R and NR subgroups, the 5-year OS rate was measured as 97% and 84%, respectively, and the PFS rate was 92% and 78%, respectively.

**Fig. 2:**
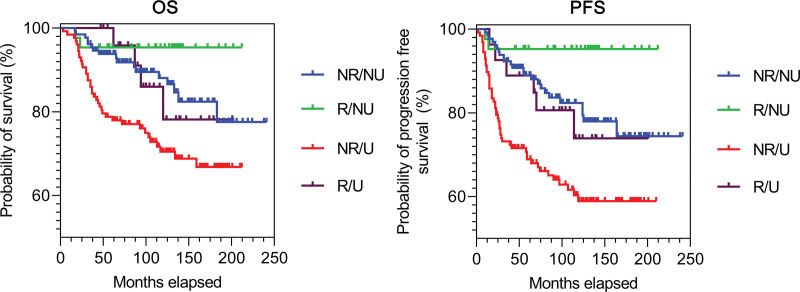
Kaplan–Meier analysis for OS and PFS in the NR/NU, NR/U, R/NU and R/U groups. NR/NU, non-regression/non-ulceration; NR/U, non-regression/ulceration; OS, overall survival; PFS, progression-free survival; R/NU, regression/non-ulceration; R/U, regression/ulceration.

The data showed similar results when we examined patients divided by Grouping 2. OS and PFS were shown to be the most favoured in the patient groups with regression but no ulceration (R/NU), while the presence of ulceration and absence of regression (NR/U) were associated with the poorest OS and PFS (Fig. 2). The HR and *P*-values calculated for the pairwise comparison: are presented in Table 2.

**Table 2:** HR and *P*-values from the comparison of the OS and PFS functions in the four patient groups

Comparison	OS		
	R/NU	NR/U	R/U
NR/NU	HR = 3.048 (CI: 1.120–8.562) *P* = 0.1089	HR = 0.4482 (CI: 0.2564–0.7475) *P* = 0.0038	HR = 0.8800 (CI: 0.2830–2.737) *P* = 0.05339
R/NU		HR = 0.1455 (CI: 0.071–0.872) *P* = 0.0020	HR = 0.2920 (CI: 0.05560–1.533) *P* = 0.1293
NR/U			HR = 2.095 (CI: 0.9516–4.610) *P* = 0.1498

	PFS		
	R/NU	NR/U	R/U
NR/NU	HR = 4.265 (CI: 1.780–10.22) *P* = 0.0312	HR = 0.4207 (CI: 0.2646–0.6689) *P* = 0.0003	HR = 0.8360 (CI: 0.3245–2.154) *P* = 0.1549
R/NU		HR = 0.104 (CI: 0.011–0.202) *P* = 0.0001	HR = 0.2059 (CI: 0.04946–0.8573) *P* = 0.0318
NR/U			HR = 1.994 (CI: 1.023–3.887) *P* = 0.1023

To calculate HR, the rows represent the numerator and the columns the denominator.

CI, confidence interval; HR, hazard ratio; NR/NU, non-regression/non-ulceration; NR/U, non-regression/ulceration; OS, overall survival; *P, P*-value; PFS, progression-free survival; R/NU, regression/non-ulceration; R/U, regression/ulceration.

### Breslow thickness is the most significant predictor of OS and PFS in all melanoma patient groups receiving IFN therapy

The Cox proportional hazard model was used to explore the effect of different predictors on OS and PFS in the four groups (Grouping 1) (Table 3). Based on the results, age and macroscopic tumour size have a minimal impact on OS and PFS among patients without regression (NR), thus increasing the risk of death and progression. Among patients with regressive melanoma (R), age and macroscopic size lose their relevant effect on OS, but macroscopic size is still a significant factor for PFS. In the NU group, neither age nor macroscopic size is a relevant predictor, whereas macroscopic size is a minimal negative predictor for OS and PFS in group U. In all four groups, Breslow thickness proved to be the most relevant negative prognostic factor for OS and PFS. Sentinel positivity was found to be significant negative prognostic factor in the Ulceration group only. The results in the other groups suggest that the effect is variable and NS.

**Table 3:** Parameters of the Cox proportional hazard model

Group	OS/PFS	Variable	B	*P*-value	HR	95% CI lower limit	95% CI upper limit
Non-regression	OS	Age	0.026	0.022	1.027	1.003	1.054
		Macroscopic size (mm^2^)	0.001	0.007	1.001	1	1.001
		Breslow (mm)	0.199	0.30	1.22	1.558	3.269
		Sentinel positivity	0.814	0.10	1.457	0.991	1.531
	PFS	Age	0.025	0.005	1.025	1.008	1.043
		Macroscopic size (mm^2^)	0.001	0.001	1.001	1	1.001
		Breslow (mm)	0.212	0	1.237	1.149	1.331
		Sentinel positivity	0.598	0.22	1.819	0.988	2.430
Regression	OS	Age	-0.58	0.252	0.943	0.864	1.042
		Macroscopic size (mm^2^)	0	0.483	0.999	0.998	1.002
		Breslow (mm)	0.686	0.003	1.43	1.267	3.112
		Sentinel positivity	-0.837	0.3	0.433	0.159	1.118
	PFS	Age	0.045	0.237	1.047	0.971	1.128
		Macroscopic size (mm^2^)	0.002	0.019	1.002	1	1.003
		Breslow (mm)	0.357	0.002	1.428	1.14	1.79
		Sentinel positivity	-0.272	0.585	0.762	0.287	2.021
Non-ulceration	OS	Age	0.035	0.106	1.035	0.933	1.080
		Macroscopic size (mm^2^)	-0.002	0.249	0.998	0. 933	1.002
		Breslow (mm)	0.375	0.001	1.455	1.178	1.789
		Sentinel positivity	0.704	0.71	2.022	0.942	4.341
	PFS	Age	0.003	0.855	1.003	0.976	1.03
		Macroscopic size (mm^2^)	0.001	0.214	1.001	1	1.002
		Breslow (mm)	0.268	0	1.308	1.14	1.5
		Sentinel positivity	0.241	0.463	1.273	0.668	2.428
Ulceration	OS	Age	0.019	0.277	1.019	0.985	1.055
		Macroscopic size (mm^2^)	0.001	0.022	1.001	1.003	1.007
		Breslow (mm)	0.14	0.009	1.15	1.036	1.276
		Sentinel positivity	0.728	0.222	2.071	0.991	3.101
	PFS	Age	0.013	0.386	1.013	0.984	1.042
		Macroscopic size (mm^2^)	0.001	0.019	1.001	1	1.007
		Breslow (mm)	0.159	0.22	1.172	1.023	1.343
		Sentinel positivity	0.487	0.01	1.627	1.178	2.248

B, regression coefficient; CI, confidence interval; HR, hazard ratio; NU, non-ulceration; OS, overall survival; PFS, progression-free survival; U, ulceration.

### The better outcome in group R is not due to lower Breslow thickness

In order to rule out the possibility that the thinner Breslow size in the R group causes more favourable outcomes, the Cox proportional hazards model was used to analyse the impact of ulceration and regression on OS and PFS with regard to Breslow thickness. For this purpose, patients were examined by Grouping 2 and stratified by Breslow thickness (<1 mm; 1.01–2 mm; 2.01–4 mm; >4 mm). In the model, all the groups were compared to the NR/NU group.

**Fig. 3:**
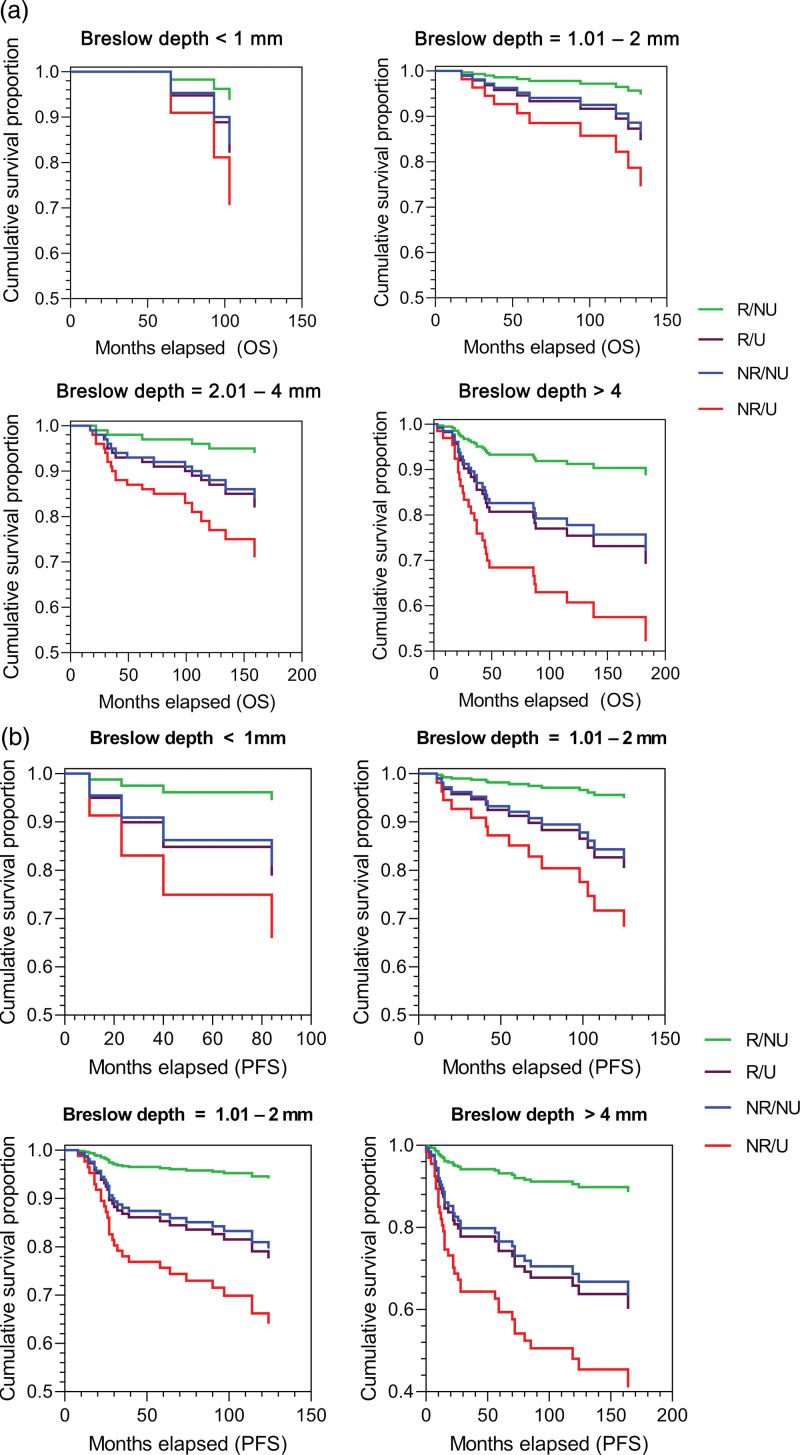
Cox regression models for OS and PFS in Grouping 2 and stratified by Breslow thickness. Panels a and b show the OS and PFS, respectively. NR/NU, non-regression/non-ulceration; NR/U, non-regression/ulceration; OS, overall survival; PFS, progression-free survival; R/NU, regression/non-ulceration; R/U, regression/ulceration.

The results of the Cox proportional hazards model equally suggest that patient outcome is the most favourable in the R/NU group in terms of both OS and PFS (HR = 0.364 and 0.280) regardless of primary tumour thickness. However, the thicker the tumour, the more significant the prognostic factor of regression (Table 4 and Fig. 3). In contrast, the presence of ulceration and the absence of regression (NR/U) are associated with a high risk of death and tumour progression (HR = 1.984 and 2.028).

**Table 4:** Parameters of Cox proportional hazards models for the impact of ulceration and regression on OS and PFS stratified by Breslow depth categories using Grouping 2

OS/PFS	Group	B	*P*-value	HR	95.0% CI for HR	
					Lower	Upper
OS	NR/U	.685	0.028	1.984	1.075	3.662
	R/NU	-1.010	0.04	.364	1.003	1.782
	R/U	.115	0.837	1.122	.374	3.369
PFS	NR/U	.706	0.007	2.025	1.209	3.392
	R/NU	-1.274	0.013	.280	1.062	1.263
	R/U	.145	0.753	1.156	.469	2.849

The groups were compared to the NR/NU group.

B, regression coefficient; CI, confidence interval; HR, hazard ratio; NR/U, non-regression/ulceration; OS, overall survival; PFS, progression-free survival; R/NU, regression/non-ulceration; R/U, regression/ulceration.

### The frequency of sentinel metastasis does not depend on either ulceration or regression

Comparing the sentinel-negative and positive column rates in the different patient groups (Grouping 1), no statistical difference in incidence was identified. Sentinel-negative lymph node status was detected: n = 110 (42%) in NR group, n = 29 (38%) in R group, n = 72 (43%) in NU group and n = 60 (40%) in U group, while sentinel positive lymph node status was detected: n = 150 (58%) in NU group, n = 48 (62%) in R group, n = 98 (57%) in NU group and n = 90 (60%) in U group.

## Discussion

The aim of the present study was to analyse the correlation between survival rate and the presence of ulceration and regression among IFN-treated melanoma patients.

As melanoma is a powerful immunogenic cancer, a strong host immune response to the tumour is believed to be the origin of the histological regression.

Adjuvant therapy for melanoma has radically changed over the past few years—anti-PD-1 or BRAF-directed therapy is the new standard of care. However, during our study period between 2000 and 2018, interferon was the only approved adjuvant therapy in melanoma patients with a high risk of recurrence after surgical resection of the primary lesion. Data conventionally suggest prolonged, relapse-free survival and overall survival; however, the optimal dose of use and duration of treatment above a toxic level have not been clarified. The mechanisms of its biologic action are generally considered to be indirect immunomodulatory effects. These are mediated through multiple molecular actions, such as promoting the Th1-mediated immune response which results in intratumoral accumulation of CD8-positive T cells and cell-mediated cytotoxicity; amplification of dendritic cell survival, maturation and antigen-presenting activity; and an antiproliferative, antiangiogenic and proapoptotic effect [11,21–23].

Many prognostic factors have been investigated and identified (various cytokines, ulceration and disease stage) for an improved selection of melanoma patients for IFN treatment; however, they have not been approved for the clinical decision-making process absent valid prospective trials [21]. Helen Gogas and colleagues found that the appearance of autoantibodies or clinical manifestations of autoimmunity during treatment with IFN alpha-2b is associated with statistically significant improvements in relapse-free survival and overall survival in patients with melanoma [24]. In 2015, they also published a review about who benefits most of adjuvant IFN therapy in melanoma. They found no association with the dose or duration of treatment and only ulceration is associated negatively with the therapy among tumour-specific factors [25]. Ives *et al*. published other results in their meta-analysis. There was no evidence that the benefit of IFN-α differed depending on duration or dose of treatment, or by gender, age, site of primary tumour, Breslow thickness, disease stage, or presence of clinical nodes, but patients with ulcerated tumours appeared to obtain benefit from IFN-α [26].

Ulceration is a well-characterised prognostic factor for melanoma patients, which is associated with an increased risk of recurrence, metastasis to the SLN and mortality [2–4,27]. Our data support a negative prognostic significance, as the presence of ulceration was associated with decreased OS and PFS among our melanoma population. Its incidence and effect on prognosis were found to be independent of the appearance of regression.

The frequency of histological regression varies in the literature, with the range reported being between 10% and 35% [28]. The melanoma population under examination presented approximately 20%. This phenomenon is accompanied by melanophages, new vessels and infiltration of variable inflammatory cells [29,30]. It can be assumed that a host immunological response to melanoma could be the cause of regression. Ma *et al*. [31] reported the presence of primary tumour histological regression results from a T cell immune response. Other data suggest that tumour-directed T-cell responses may play a role in an improved prognosis as shown by successful treatment with oncological therapies stimulating the responsiveness of the host immune system [32–35].

It is unknown whether the presence of spontaneous regression in melanoma can predict response to immunotherapy. On the other hand, clear links between the two phenomena have been demonstrated clinically and immunologically too, in a case of vitiligo-like depigmentation in melanoma patients. Vitiligo is an autoimmun disorder in witch melanocytes are destroyed, leading to depigmented patches of skin. It has a common immunological basis with spontaneous regression: infiltrate of CD8 + T cells against melanocyte/melanoma-shared antigens, which serve as immune targets, and decreased infiltrate of Treg subsets [36,37]. In melanoma patients, depigmented patches has been reported as an immune-related adverse effect of checkpoint blockade furthermore it has been shown to be associated with better treatment outcomes [38,39].

Hence, we hypothesised that regression as an immunological process may have an effect on the efficacy of IFN treatment.

We found that regressive melanoma is associated with increased OS and PFS. It can thus be presumed to be a positive prognostic factor. Examining the two histological features simultaneously, we detected that the most favourable prognosis is related to patients with regression but no ulceration, while the poorest prognosis fell within the non-regressive/ulcerated group, regardless of Breslow thickness.

Owing to the various inconsistencies in assessing regression, its biologic and prognostic significance has not been clarified. As our data suggest, there are some recent studies that have also reported regression as a favourable characteristic in cutaneous melanoma. Ribero *et al*. found that it plays a protective role in overall survival and disease-free survival of stage I–II melanoma patients with histological regression (American Joint Committee on Cancer stages I–II); in addition, regression alone should not be a reason to perform SLNB in thin melanoma [19]. Zunga *et al*. investigated regression in stage III positive SLN melanoma patients and reported that histological regression (n = 43) was associated with a better prognosis (sub-HR = 0.34, CL 0.12–0.92) [9].

The most important prognostic factor in intermediate and thick melanoma is sentinel lymph node (SLN) positivity [30,40–42]. Nevertheless, there is no uniform consensus on the link between regression and lymph node metastasis. Some early studies reported that thin melanomas with regression were more prone to metastasis [15,43], while others found no relation [44–46]. A recent study demonstrated that histological regression is associated with negative SLN and that there is no relation between regression and patient outcome, with survival influenced by a Breslow thickness of >2 mm and a positive SLN status [47]. Some authors have reported that the presence of regression may be associated with lower rates of SLN metastasis [48,49]. In this study, we calculated the SLN metastasis rate, and we found no significant link to regression or ulceration.

Our results support our hypothesis, as it allows us to conclude that regression does not imply a head start when melanoma is detected, but advantageous processes emerge in regressive melanoma during IFN therapy. Regression is a mechanism of immune function, thus theoretically, patients with regressive melanoma might obtain more benefit from adjuvant immun therapy than target therapy. This results in an improved therapeutic decision-making process.

In conclusion, we have demonstrated a significant relation not only between ulceration and the outcomes of patients with malignant melanoma but also between regression and these outcomes. Our data suggest that the presence of regression seems to be a positive prognostic factor among IFN-treated patients. However, we cannot assert that regression has an impact on the adjuvant therapy itself, owing to the fact that our entire patient population received this therapy and we did not compare it to a control group without any systemic treatment. Hence we couldn’t exclude that regression could be advantageous in itself, without any therapy.

A strength of the study is that the same established team conducted the work during the period under examination based on consistent principles. Distortions from subjective factors in setting up the histopathological parameters can be considered minimal. However, one limitation in evaluating the results is the retrospective analysis and the relatively low number of members in particular subgroups. On the basis of our observations, it is clear that an express evaluation of the clinical picture of primary melanoma continues to provide an important message for the clinician. Beyond the macroscopic spread, signs of regression may offer support in making a more accurate prognosis.

Our results suggest that, besides the mutation status, a simple histological marker such as regression can also be taken into account when considering which therapy to choose for a melanoma patient in order to achieve the most effective treatment. The validation of the data naturally requires further analyzes in the case of our patients who received innovative therapy.

## Acknowledgements

### Conflicts of interest

There are no conflicts of interest.
